# Learn the interactions: Weakly supervised video anomaly detection with human-object interactions

**DOI:** 10.1371/journal.pone.0358538

**Published:** 2026-09-18

**Authors:** Maowen Zhou, Erma Rahayu Mohd Faizal Abdullah, Aznul Qalid Md Sabri, Susanto Rahardja

**Affiliations:** 1 Department of Artificial Intelligence, Universiti Malaya, Kuala Lumpur, Wilayah Persekutuan Kuala Lumpur, Malaysia; 2 Engineering Cluster, Singapore Institute of Technology, Singapore, Singapore; Instituto Politecnico Nacional, MEXICO

## Abstract

Weakly supervised video anomaly detection remains a challenging problem, primarily due to the scarcity of abnormal training samples and the lack of diverse feature representations, which hamper the learning of discriminative models. To address these issues, we introduce a novel weakly supervised cross-domain framework that enriches feature representations by explicitly integrating semantic cues from Human-Object Interactions (HOI) and action dynamics. Specifically, we model domain-specific features using two independent encoders and fuse them via a bi-directional cross-attention mechanism to obtain a unified embedding. To further alleviate class imbalance and enhance feature discrimination, our network is jointly optimized with classification loss, a cross-domain contrastive loss, and a clip-level focal loss. The proposed method is evaluated with various visual backbones, demonstrating its adaptability and robustness. Extensive experiments show that our approach achieves competitive performance against state-of-the-art methods under weak supervision in terms of AUC. Specifically, our proposed method achieves 98.33% AUC and 87.96% AUC on two benchmark datasets, i.e., ShanghaiTech and UCF-Crime, respectively.

## Introduction

The task of video anomaly detection (VAD) aims to automatically identify the starting and ending frames of any anomalous event that is different from previously learned normal representation in a video and maintain continuous detection throughout the duration of the anomalous event [1–15]. Typical anomalous events include traffic accidents, fights, thefts, and arson. Recent research in VAD falls into three primary paradigms: fully-supervised VAD [5], unsupervised VAD (UVAD) [16–22], and weakly supervised video anomaly detection (WSVAD) [1–15,23,24]. Among these, WSVAD has become increasingly popular owing to its practical scalability. Frame-level annotations are prohibitively costly for long, untrimmed surveillance videos, whereas WSVAD methods typically require only coarse video-level labels and are often based on Multiple Instance Learning (MIL).

**Fig 1:**
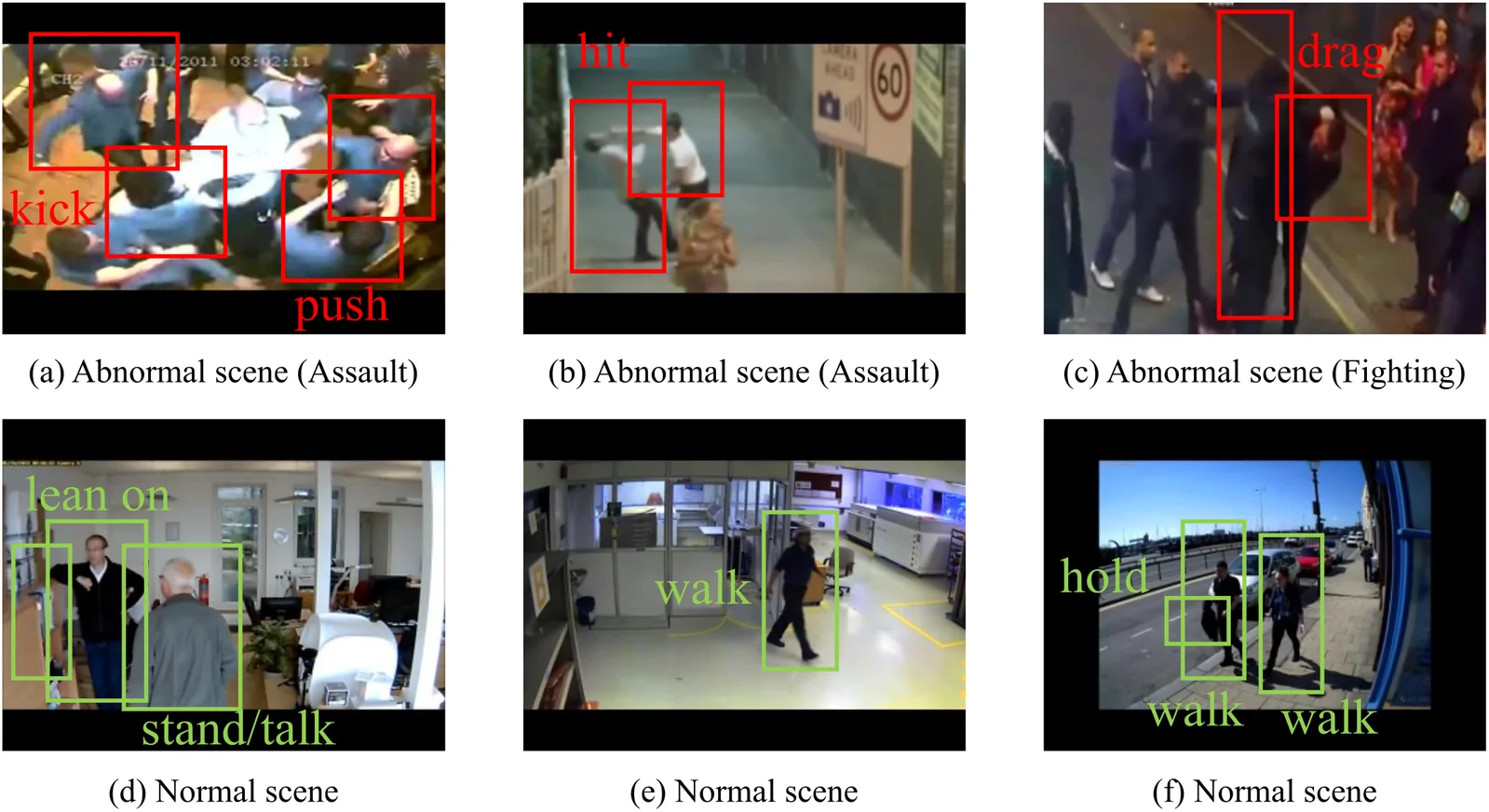
Motivational illustration of <*human, interaction, object*> triplets for anomaly discrimination. The images are taken from UCF-Crime [6]. The figure highlights the discriminative power of these HOI cues in distinguishing anomalous from normal events. These interaction-based representations capture fine-grained relational cues that are important for identifying human-related abnormal events, yet often overlooked by motion features alone. In abnormal scenes (a–c), high-impact interactions such as *kick*, *hit*, *push*, and *drag* typically co-occur with strong motion patterns. In contrast, normal scenes (d–f) involve benign interactions such as *talk* or *stand*, which are often accompanied by subtle motion patterns. This motivates our cross-domain learning strategy, which incorporates HOI features to complement motion-based representations and enhance anomaly discrimination.

Although existing methods [1–15,23,24] have demonstrated promising performance on public benchmarks, they still suffer from three primary limitations. 1) *Unbalanced feature learning.* In real-world surveillance scenarios, WSVAD is inherently affected by severe class imbalance, where normal frames vastly outnumber anomalous ones. For instance, the testing set of UCF-Crime [6,9,13,14] contains 1,029,755 normal frames versus only 84,389 abnormal frames, and the training set includes over 12 million frames without precise frame-level labels. This class imbalance causes the latent feature space to be dominated by normal patterns, making it difficult for models to learn effective representations of anomalous events. Furthermore, as most frameworks adopt fixed pre-trained feature extractors during the feature extraction stage, this imbalance is inherently preserved and further propagated in the feature aggregation phase, exacerbating the problem of unbalanced feature learning. 2) *Lack of semantic diversity and complexity.* Previous frameworks typically rely on features derived from a single feature space, such as C3D [25] and I3D [26], or transformer-based networks [1,3] like VideoSwin [27], to characterize motion dynamics. In this work, the term “domain” refers to distinct feature spaces used for representation learning. While these single-domain features effectively capture temporal motion cues, they often fail to encode the fine-grained semantics of human-object interactions that are critical for detecting complex anomalies in surveillance videos. Many anomalous behaviors, such as assault, fighting, or threats, involve subtle yet meaningful interactions between humans and objects—e.g., hit, kick, drag, and push—which are not fully represented by motion-centric action features alone. As illustrated in Fig 1, a great portion of anomalous events in datasets like UCF-Crime [6] involve clear human-object interactions, such as a person pushing another or engaging in physical altercations. Without explicit modeling of such interactions, models are prone to confusing benign scenes like people walking or conversing with genuinely anomalous ones involving violent behavior, especially when abnormal frames are scarce. 3) *Insufficient supervision signals.* Most WSVAD frameworks are trained with only video-level labels, lacking precise frame- or clip-level annotations. This weak supervision makes it challenging for models to accurately localize anomalous events, particularly when they are temporally sparse or short-lived. These limitations collectively reflect one fundamental challenge in the current weakly supervised setting: the lack of training data, which hinders the model from learning discriminative representations. To address this challenge, it becomes crucial to incorporate various complementary semantic information to enhance the discriminative power of the learned features.

Specifically, we propose VADHOI (Video Anomaly Detection with Human-Object Interactions), a novel weakly supervised framework specifically designed with a cross-domain feature learning strategy. Central to this strategy is the Human-Object Interaction and Action Learning (HOIAL) module that captures spatio-temporal motion cues and human-object semantics through two domain-specific encoders. These features are subsequently fused using a bi-directional cross-attention mechanism, allowing the model to learn a unified representation that reflects both motion and interaction cues. The proposed cross-domain representation framework not only enriches semantic diversity but also helps alleviate class imbalance from a representational standpoint. By incorporating multiple feature domains, the model learns from a broader and more varied embedding space, making it less susceptible to bias toward dominant normal patterns. Moreover, as feature extractors are fixed during the feature extraction stage, the opportunity to manipulate feature representations is inherently constrained. Therefore, we address class imbalance at the feature aggregation stage, where adaptive weighting and domain-aware integration can be more effectively performed in a task-specific and learnable manner.

To further support effective learning under weak supervision, we introduce two novel loss functions. The first is a Cross-Domain Contrastive Loss, which encourages intra-class compactness and inter-class separability across both the motion and interaction domains, thereby facilitating more discriminative boundaries in the joint embedding space. The second is a Clip-Level Focal Loss, which explicitly addresses class imbalance by assigning greater importance to hard-to-classify clips while reducing the weight of well-classified ones. To support this, clip-level pseudo-labels are generated using a state-of-the-art WSVAD model [4] and refined through temporal smoothing to enhance their consistency and reliability. Together, these loss functions provide stronger supervision signals at the clip level, leading to improved model robustness and more precise anomaly localization.

Finally, the fused cross-domain features are processed by a Multi-scale Temporal Network (MTN) [9] that combines a temporal self-attention module and a pyramid of dilated convolutions. This enables the model to effectively capture both short- and long-range dependencies, further enhancing its ability to detect temporally sparse or subtle anomalies. Extensive experiments on UCF-Crime [6] and ShanghaiTech [16,17] demonstrate the superior performance and robustness of our framework compared to state-of-the-art methods.

In a nutshell, our contributions are summarized as follows:

- We propose **VADHOI**, a novel WSVAD framework that incorporates cross-domain and multi-scale features to enrich semantic diversity. By fusing HOI and action features, our method effectively addresses the limitations arising from insufficient information within a single domain. To the best of our knowledge, this is the first WSVAD approach to explicitly exploit cross-domain semantics in this way.
- We introduce a **cross-domain contrastive loss** and a **clip-level focal loss** to address class imbalance and improve the separability of anomalous patterns during feature aggregation and scoring.
- Extensive experiments on UCF-Crime and ShanghaiTech demonstrate that VADHOI delivers performance comparable to state-of-the-art methods, achieving strong accuracy and robustness across both datasets. The code will be made available at https://github.com/MW-ZHOU/VADHOI.

## Related works

### Weakly supervised video anomaly detection

It is widely recognized that annotating every video frame in the training set is highly time-consuming and impractical. Therefore, relevant studies on VAD focus mainly on two settings: unsupervised learning [16–22,28,29] and weakly supervised learning [1–7,9–13,15,23,24,30]. UVAD learns representations with normal videos only and then identifies frames with large prediction errors [16,22] or large reconstruction errors [17–19,21] as anomalous. The main limitations of UVAD frameworks are the lack of any prior knowledge of true anomalies and their suboptimal assumptions on prediction and reconstruction errors.

The mainstream of current research is WSVAD, which uses training data annotated at the video level. WSVAD methods are primarily built upon the MIL framework [6], assuming that the anomaly score of an abnormal frame is higher than that of a normal frame. MIL-based methods treat each video as a bag containing multiple video segments/clips, and the clip with the highest anomaly score is chosen to represent the whole video. Multiple methods tried to [1,3,4,9] enhance the robustness of the top-1 ranking loss by aggregating video clips with the top-*k* largest anomaly scores. Tian et al. [9] used the feature magnitude (i.e., *l*_2_ norm) of a video clip as a measure of abnormality, based on the assumption that the feature magnitude of an abnormal clip is larger than or equal to the feature magnitude of a normal clip. Some works [1,13] attempted to find an alternative abnormality criterion because clip-level feature magnitude often overlooks the difference between anomalous events of distinct scenes. Zhou et al. [13] used the Divergence of Feature from Mean (DFM) vector of BatchNorm as a measure of abnormality to reduce the impact of noisy labels. Recently, some works drew inspiration from prompt learning and leveraged pre-trained vision-language models to improve context and semantic modeling [14,31]. Tan et al. [8] combined a transformer-based classifier with existing frameworks such as RTFM [9] using whole-video classification as the supervision signal. Recently, there emerged some works that attempted to leverage the power of Visual-Language Pretraining, such as using multi-modal features extracted by the fixed image and text encoder of CLIP [14,31,32].

### Self-training

Self-training has been utilized in WSVAD [2,3,7,10,15,33] and UVAD [28,29]. Self-training methods expand the labeled dataset by generating pseudo-labels for unlabeled samples, allowing the model to leverage information from labeled and unlabeled sources. In WSVAD, Feng et al. [2] initially trained a pseudo-label generator and subsequently fine-tuned a task-specific encoder based on the generated pseudo-labels. Li et al. [3] proposed to use an optimization unit containing multiple instances, focusing on refining the abnormal scores with a self-training strategy as well as a multi-sequence ranking loss. Shi et al. [7] proposed an abnormal ratio-based MIL loss and a multi-phase self-training paradigm. Yang et al. [15] proposed an attention-driven self-training framework for WSVAD to address the incompleteness and noise of pseudo-labels in conventional two-stage self-training methods. Their dual-branch framework synchronizes pseudo-label generation and self-training, where the first branch introduces a video snippet separation and fusion (VSSF) module and an attention-driven pseudo-label generation module with mean-variance-based denoising to produce more reliable snippet-level pseudo-labels, while the second branch uses these pseudo-labels and employs a multi-scale temporal feature interaction learning module to train the snippet classifier. The objective of self-training is to iteratively train a model that can generate reliable pseudo-labels. In contrast, our goal is not to refine pseudo-label generation itself, but to learn interaction-aware anomaly representations by integrating HOI semantics with action features. As shown in Fig 3, instead of inferring snippet-level pseudo-labels from internally learned attention weights as in Yang et al. [15], our framework obtains clip-level anomaly scores from a pretrained VAD model and converts them into clip-level pseudo-labels through threshold-based filtering. To further reduce the impact of pseudo-label imbalance, we introduce a cross-domain focal loss [34] to work with the cross-domain contrastive loss.

### VAD with object detection and relation modeling

Recent progress in VAD has demonstrated that learning good feature representations is essential to effectively distinguish abnormal from normal events. Previous VAD approaches [1–7,9–12] often extract feature representations focused on spatial-temporal actions or motions. However, these methods neglect human-object interactions and object-wise relationships, which possess crucial semantics in real-world scenarios. For instance, a person simply standing next to a motorcycle may be classified as normal, while the more subtle action of a person standing next to a motorcycle attempting to steal it can be missed due to its nuanced nature. Several attempts have been made to address this limitation [35–39], emphasizing the growing importance of semantic modeling and structured inductive biases in VAD. For instance, Hoi2Threat [37] leverages human–object interaction cues to build an interpretable threat detection framework, PI-VAD [38] proposes a WSVAD framework that enriches RGB features with complementary cues from multiple modalities (pose, depth, panoptic masks, optical flow, and language) via pseudo modality generation and cross-modal induction to achieve more robust anomaly detection, and VTD-CLIP [39] presents a video-to-text discretization scheme that transforms continuous video features into semantically meaningful text-aligned representations via a CLIP-based learnable codebook to enhance semantic understanding in general video recognition. Öztürk et al. [36] attempted to encode action features of I3D and object features of an Object Detector (e.g., Faster RCNN) with a transformer encoder [40] and the encoder of an Object Relation Transformer [41], respectively. Then cross-attention is used to fuse the features of these two encoders. Unlike their method, which represents object-wise relations through the relative positions of object bounding boxes and object features with self-attention, our framework leverages human-object interactions for a more comprehensive understanding of the scene. Majhi et al. [35] introduced a framework that simultaneously learns human-centric and scene-centric representations using two independent subnets, combined with a soft-selection coupler that adaptively focuses on one of these two representations. Recent studies have also explored advanced interaction-aware, structure-aware, and cross-modal representation learning in related visual understanding tasks. Han et al. [42] proposed SMPL, an HOI detection model based on vision-language models that combines multi-granularity visual and textual prompts with soft-label supervision to improve generalization to rare and unseen interactions. Zhang et al. [43] proposed ASCFormer for 3D object detection, which employs an adaptive structure-aware cascaded transformer to learn discriminative representations from sparse point clouds. Jia and Zhang [44] introduced DeHub for text-video retrieval, which learns robust cross-modal representations by mitigating the hubness problem. Although these methods address different visual understanding tasks, they demonstrate the importance of interaction, structural, and cross-modal information for learning discriminative representations. We believe that enhancing the understanding of HOI is an inevitable and crucial step on the path of the development of VAD.

## The proposed method

### Overview

**Fig 2:**
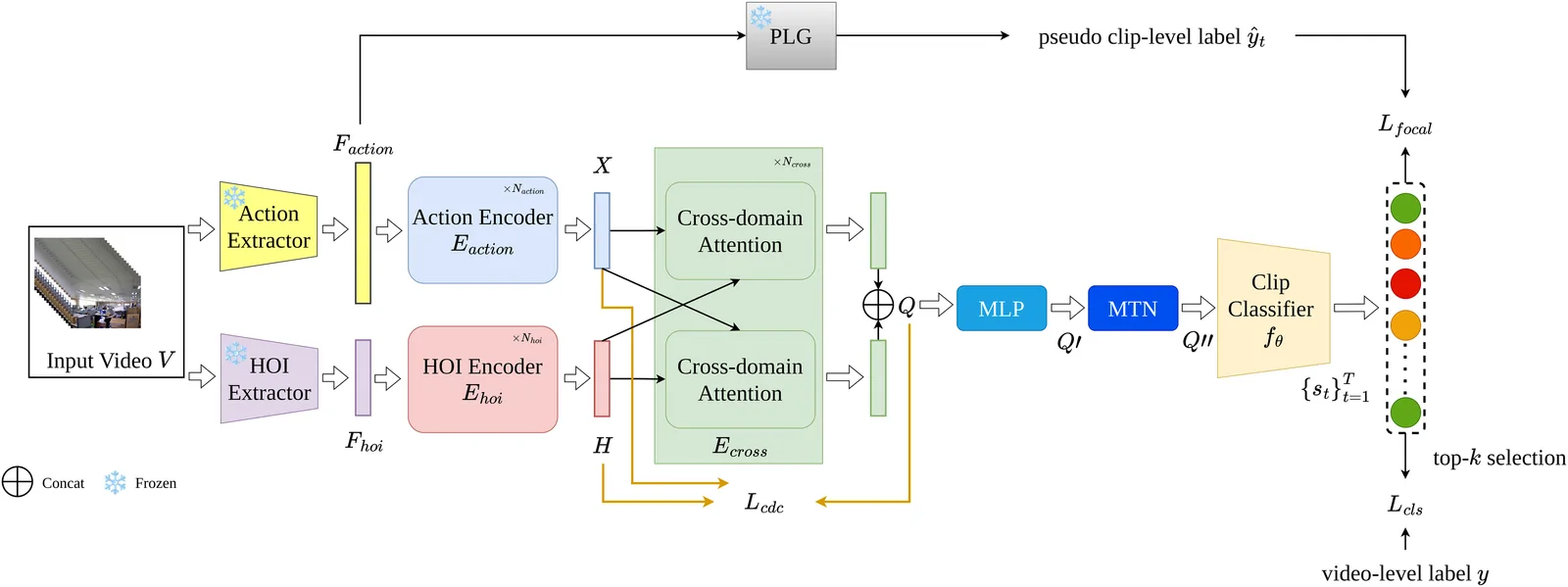
Structural overview of the VADHOI framework. To capture rich semantics and address class imbalance, VADHOI adopts a cross-domain learning strategy that integrates motion and interaction cues. Specifically, we extract action features $F_{action}$ and HOI features $F_{hoi}$ using frozen pre-trained models. These features are processed by two domain-specific encoders with self-attention to learn temporal representations. The outputs are then fused through a cross-domain encoder using bi-directional cross-attention, yielding a joint representation *Q*. This is followed by an MLP and an MTN to model temporal dynamics. A clip-level classifier $f_{\theta }$ predicts anomaly scores for each clip. The training objectives include the video-level classification loss $L_{cls}$, cross-domain contrastive loss $L_{cdc}$, and cross-domain focal loss $L_{focal}$.

Given a weakly-labeled video $V=\{v_{t}{\}}_{t=1}^{T}$ consisting of *T* non-overlapping clips, our objective is to assign an anomaly score $s_{t}\in [0,1]$ to each clip using only a video-level binary label y∈{0,1}, where *y* = 1 denotes an abnormal video and *y* = 0 denotes a normal one. To address the lack of semantic diversity caused by limited training data, we extract clip-level features from two domains: action features $F_{action}$ using a pre-trained visual backbone, and HOI features $F_{hoi}$ using a pre-trained HOI detector. These features are independently processed by two domain-specific encoders with self-attention to capture action-level and interaction-level representations. A cross-domain encoder then integrates the two domains through a bi-directional cross-attention mechanism, allowing contextual information from one domain to inform the representation in the other. This yields a unified joint representation that captures both semantic and relational cues. The fused representation is then projected by a multi-layer perceptron (MLP) and passed through the MTN to model both short- and long-range temporal dependencies at various temporal scales. Finally, a lightweight classifier predicts the clip-level anomaly scores. The overview of the VADHOI framework is shown in Fig 2.

### Human-object interaction and action learning

Existing WSVAD methods typically rely on single-domain encoders to extract action-centric features from videos. Although effective in capturing basic motion patterns, these approaches face challenges in dealing with imbalanced training data (where normal frames vastly outnumber anomalies) and limited semantic expressiveness, which hinders the learning of robust, discriminative representations. Recent successes in cross-modal representation learning, such as visual question answering (VQA) [45,46], motivate us to introduce cross-domain representation learning to WSVAD. Consequently, we propose the Human-Object Interaction and Action Learning (HOIAL) module, comprising three carefully designed encoders: an Action Encoder $E_{action}$, an HOI Encoder $E_{hoi}$, and a Cross-domain Encoder $E_{cross}$, each addressing specific challenges.

**Action Encoder** To extract motion-centric clip-level representations, we utilize one of two types of pre-extracted visual features: I3D [1,4,9,26] or **CLIP-V** [32]. I3D is a widely adopted 3D convolutional model pretrained on Kinetics-400, while **CLIP-V** visual features are extracted solely from the visual encoder of CLIP, without incorporating textual information. These CLIP features are extracted at the frame level and temporally aggregated into clip-level representations. Both types of features are integrated into our framework to assess its adaptability across different backbone architectures. Unlike VadCLIP [31], which relies on both the image and text encoders of CLIP to perform vision-language alignment, our method solely uses the image branch without any textual supervision, prompts, or multimodal pretraining. This ensures that our model remains fully vision-based and compatible with standard weakly supervised anomaly detection (WSVAD) settings.

Let $F_{action}\in {\mathbb{R}}^{P\times T\times D^{\prime }}$ denote the extracted action-domain features, where *T* is the number of video clips, *P* is the number of crops per clip, and $D^{\prime }$ is the feature dimension, which depends on the backbone type: $D^{\prime }=D$ for I3D and $D^{\prime }=\frac{D}{4}$ for **CLIP-V**. To unify the feature dimensions, a linear projection is applied to the I3D features to reduce their dimensionality from *D* to $\frac{D}{4}$, while **CLIP-V** features are used directly without modification. The resulting features are then processed by a transformer-based encoder $E_{action}$, which consists of $N_{action}$ layers, each comprising a multi-head self-attention mechanism and a feed-forward sub-layer. The encoder aims to capture long-range temporal dependencies and produce context-aware clip-level representations. The output is defined as:


$$\begin{array}{c} X=E_{action}(F_{action}),\;X\in {\mathbb{R}}^{P\times T\times \frac{D}{4}}. \end{array}$$ (1)

**HOI Encoder** Although action-based features effectively capture overall motion dynamics, they often fail to detect subtle anomalies involving specific HOI, such as suspicious behaviors characterized by subtle interactions (e.g., pushing, grabbing, hitting). Therefore, it is essential to explicitly model interaction semantics alongside action cues.

To address this semantic limitation, the HOI Encoder leverages the latent HOI features extracted by the interaction transformer of HOTR [47], pre-trained on the HICO-DET dataset [48]. The extracted HOI features $F_{hoi}\in {\mathbb{R}}^{P\times T\times \frac{D}{8}}$ encapsulate rich semantic information about pairwise relationships between humans and objects within each clip. Since the original HOI feature dimension is $\frac{D}{8}$, we apply a linear projection to increase it to $\frac{D}{4}$ to ensure dimensional consistency with other feature branches. The transformed features are then passed through a transformer-based encoder $E_{hoi}$, which models the temporal evolution of interaction patterns across the video sequence:


$$\begin{array}{c} H=E_{hoi}(F_{hoi}),\;H\in {\mathbb{R}}^{P\times T\times \frac{D}{4}}, \end{array}$$ (2)

where *H* represents the encoded HOI features. Similar to the action encoder, $E_{hoi}$ comprises $N_{hoi}$ transformer layers to model long-range interactions, enhancing the sensitivity of the framework to anomalies involving subtle or complex interactions. We apply 10-crop augmentation when extracting HOI representations, following the conventional feature extraction strategy used for latent I3D-RGB features in WSVAD. The extracted HOI features are not explicitly assigned as normal or abnormal; instead, their anomaly-discriminative patterns are learned jointly with action features under weak supervision.

While recent human-object interaction (HOI) detectors such as GEN-VLKT [49], HOICLIP [50], and SMPL [42] have shown better accuracy on HOI benchmarks, they often require large-scale fine-tuning and incur substantial computational overhead. In contrast, HOTR [47] provides a favorable trade-off between accuracy and efficiency, enabling us to extract HOI features with acceptable latency and resource usage. Given that our framework needs to process long, untrimmed surveillance videos with limited supervision, HOTR [47] serves as a practical and effective solution. Moreover, the proposed HOIAL module is inherently model-agnostic and can be easily extended to accommodate stronger HOI detectors in future work without architectural modifications.

**Cross-domain Encoder.** While action and HOI encoders independently provide valuable semantic cues, their isolated usage can lead to misaligned or inconsistent representations. Without explicitly modeling inter-domain relationships, the complementary nature of these two feature spaces remains underexploited, potentially causing anomalies to be obscured by dominant normal patterns.

To solve this alignment and fusion problem, we propose the Cross-domain Encoder $E_{cross}$. Its primary objective is to bridge the semantic gap between the action and HOI domains, producing unified and discriminative joint representations. Specifically, $E_{cross}$ incorporates bi-directional cross-attention modules, which allow action and HOI features to mutually condition and align with each other. For each clip t∈[1,T], we denote the action and HOI features as $x_{t}^{n-1}\in {\mathbb{R}}^{D/4}$ and $h_{t}^{n-1}\in {\mathbb{R}}^{D/4}$, respectively, where n is the index of the cross-domain encoder layer, and the feature dimension is already reduced by prior processing. In each of the *N*_cross_ layers, the encoder employs bi-directional cross-attention so that action features can attend to HOI features and vice versa. Specifically, for the *t*-th clip at layer *n*:


$$\begin{array}{c} {\hat{x}}_{t}^{n}=CrossAtt(x_{t}^{n-1},\{h_{1}^{n-1},...,h_{T}^{n-1}\}), \end{array}$$ (3)

$$\begin{array}{c} {\hat{h}}_{t}^{n}=CrossAtt(h_{t}^{n-1},\{x_{1}^{n-1},...,x_{T}^{n-1}\}). \end{array}$$ (4)

These cross-attended representations are then refined via self-attention within each domain to enhance internal semantic consistency:


$$\begin{array}{c} {\tilde{x}}_{t}^{n}=SelfAtt({\hat{x}}_{t}^{n-1},\{{\hat{x}}_{1}^{n-1},...,{\hat{x}}_{T}^{n-1}\}), \end{array}$$ (5)

$$\begin{array}{c} {\tilde{h}}_{t}^{n}=SelfAtt({\hat{h}}_{t}^{n-1},\{{\hat{h}}_{1}^{n-1},...,{\hat{h}}_{T}^{n-1}\}). \end{array}$$ (6)

Each of these attention modules is complemented by a feed-forward network with residual connections and layer normalization, ensuring stable representation learning. After the final cross-domain encoder layer, the clip-level features from both domains are concatenated for each clip to form a unified representation:


$$\begin{array}{c} q_{t}=[{\tilde{x}}_{t}^{N\text{cross}};{\tilde{h}}_{t}^{N\text{cross}}],\;q_{t}\in {\mathbb{R}}^{\frac{D}{2}}, \end{array}$$ (7)

resulting in a feature matrix $Q\in {\mathbb{R}}^{T\times \frac{D}{2}}$ for a video.

To reduce dimensionality and unify the representation space, we further apply a lightweight multi-layer perceptron (MLP) that projects *Q* to a lower-dimensional space:


$$\begin{array}{c} Q^{\prime }=\text{MLP}(Q),\;Q^{\prime }\in {\mathbb{R}}^{T\times \frac{D}{4}}. \end{array}$$ (8)

This simple yet effective cross-domain fusion step alleviates representational bias, maximizes the complementary strengths of both domains, and ultimately enables more accurate and robust anomaly detection under weak supervision.

### Multi-scale temporal network

As demonstrated in previous WSVAD studies [4,9], capturing both global and local temporal dependencies at multiple temporal resolutions is critical for accurate VAD. While most existing methods model temporal dynamics within a single feature domain, our framework leverages a joint cross-domain representation that encodes complementary semantics from both action and human-object interaction (HOI) domains.

Concretely, the output of the HOIAL module consists of two domain-specific feature sequences: one from the action branch and the other from the HOI branch. These features are first concatenated along the feature dimension and then projected into a unified embedding space via a linear transformation to produce the cross-domain joint representation $Q^{\prime }$. This operation not only preserves the semantic richness of both domains but also aligns their dimensionality for subsequent temporal modeling. The joint representation $Q^{\prime }$ is then passed to the MTN, which produces $Q^{\prime \prime }$ by capturing both long-range and short-range temporal dependencies at various temporal scales. The MTN comprises a temporal self-attention (TSA) module, which models global temporal relations by attending across the entire sequence, and a pyramid of dilated convolutions (PDC), which captures local temporal context at multiple scales via dilated temporal convolutions. Finally, the temporally enriched cross-domain representation is fed into a lightweight clip-level classifier $f_{\theta }$ to produce anomaly scores for each video clip.

### Clip-level pseudo label generation

**Fig 3:**
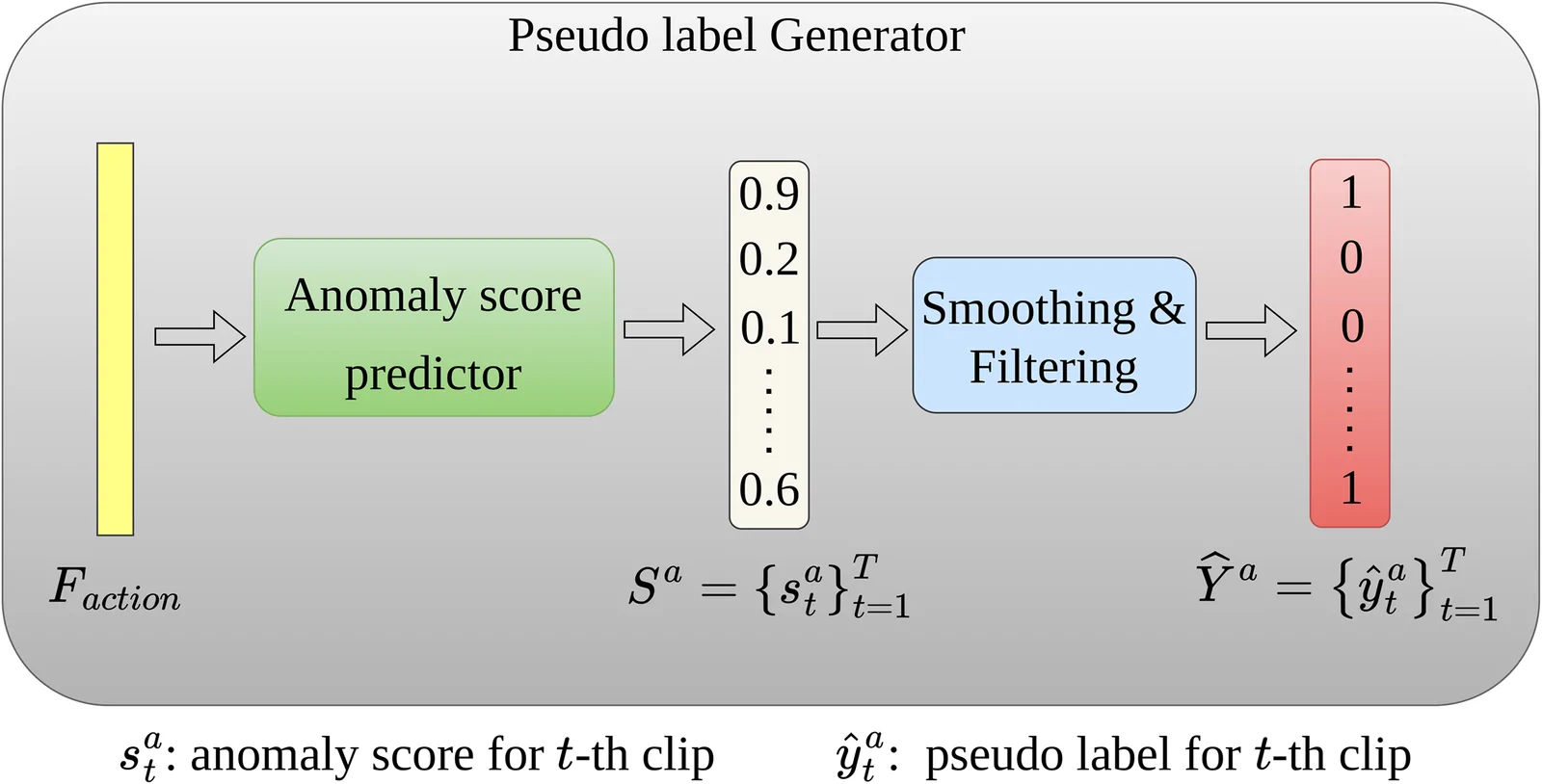
Clip-level pseudo label generation. A pre-trained VAD model is used to obtain clip-level pseudo labels.

In the training videos, the vast majority of frames are normal, leading to a severe imbalance between the number of normal and abnormal frames. This imbalance persists after extracting clip-level features using the pre-trained backbone network. To address the imbalance between normal and abnormal clips, we propose a novel clip-level focal loss in conjunction with pseudo clip-level labels. Since all clips within a normal video $V^{n}$ are normal, we set pseudo clip-level labels for all these clips to 0, i.e., ${\hat{Y}}^{n}=\{{\hat{y}}_{t}^{n}{\}}_{t=1}^{T}$, where ${\hat{y}}_{t}^{n}=0$. We leverage a pre-trained model [4] as a pseudo label generator (PLG) to generate pseudo clip-level labels for all abnormal videos only. As shown in Fig 3, PLG takes I3D-RGB features $F_{action}$ extracted from an abnormal video $V^{a}$ as input and outputs clip-level anomaly scores $S^{a}=\{s_{t}^{a}{\}}_{t=1}^{T}$. Following the temporal smoothing strategy commonly adopted in WSVAD [2,14], we apply a mean filter with kernel size k to smooth the predicted anomaly scores over clips within a local temporal window centered at clip *t*:


$$\begin{array}{c} {\tilde{s}}_{t}^{a}=\frac{1}{2k}\sum_{j=t-k}^{t+k}s_{j}^{a},t\in [1,T]. \end{array}$$ (9)

The objective here is to reduce the jitter in anomaly scores, resulting in more consistent predictions with reduced false alarms. Then we transfer smoothed anomaly scores $\{{\tilde{s}}_{t}^{a}{\}}_{t=1}^{T}$ into binary pseudo labels ${\hat{Y}}^{a}=\{{\hat{y}}_{t}^{a}{\}}_{t=1}^{T}$, ${\hat{y}}_{t}^{a}\in \{0,1\}$ with:


$$\begin{array}{c} {\hat{y}}_{t}^{a}=\left\{ \begin{array}{cc} 1, & \text{if }{\tilde{s}}_{t}^{a}\geq med({\tilde{s}}_{t}^{a})\\ 0, & \text{otherwise} \end{array}\right.,t\in [1,T], \end{array}$$ (10)

where med(*) is the median of the anomaly scores of clips within each video. These pseudo clip-level labels ${\hat{Y}}^{a}$ and ${\hat{Y}}^{n}$ are used in cross-domain focal loss that is defined by Eq (17).

### Loss function

Following [1,4,6,9], we take advantage of the top-*k* feature magnitude learning presented. In detail, clip-level anomaly scores are computed with $f_{\theta }(Q^{\prime \prime })=\{s_{t}{\}}_{t=1}^{T}$, and video-level anomaly score *s* is the average anomaly scores of the top-3 clips with the highest joint feature magnitudes. The clip classifier $f_{\theta }$ is trained with a standard Binary Cross-Entropy loss:


$$\begin{array}{c} {ℒ}_{cls}=-(ylog(s)+(1-y)log(1-s)), \end{array}$$ (11)

where *y* is video-level label.

**Cross-domain contrastive loss** In WSVAD, learning discriminative representations is especially challenging due to the severe imbalance between normal and abnormal clips. This often leads to collapsed feature distributions, where rare anomalies are poorly separated from the dominant normal patterns, particularly when features from different semantic domains (e.g., action vs. interaction) are aggregated.

**Fig 4:**
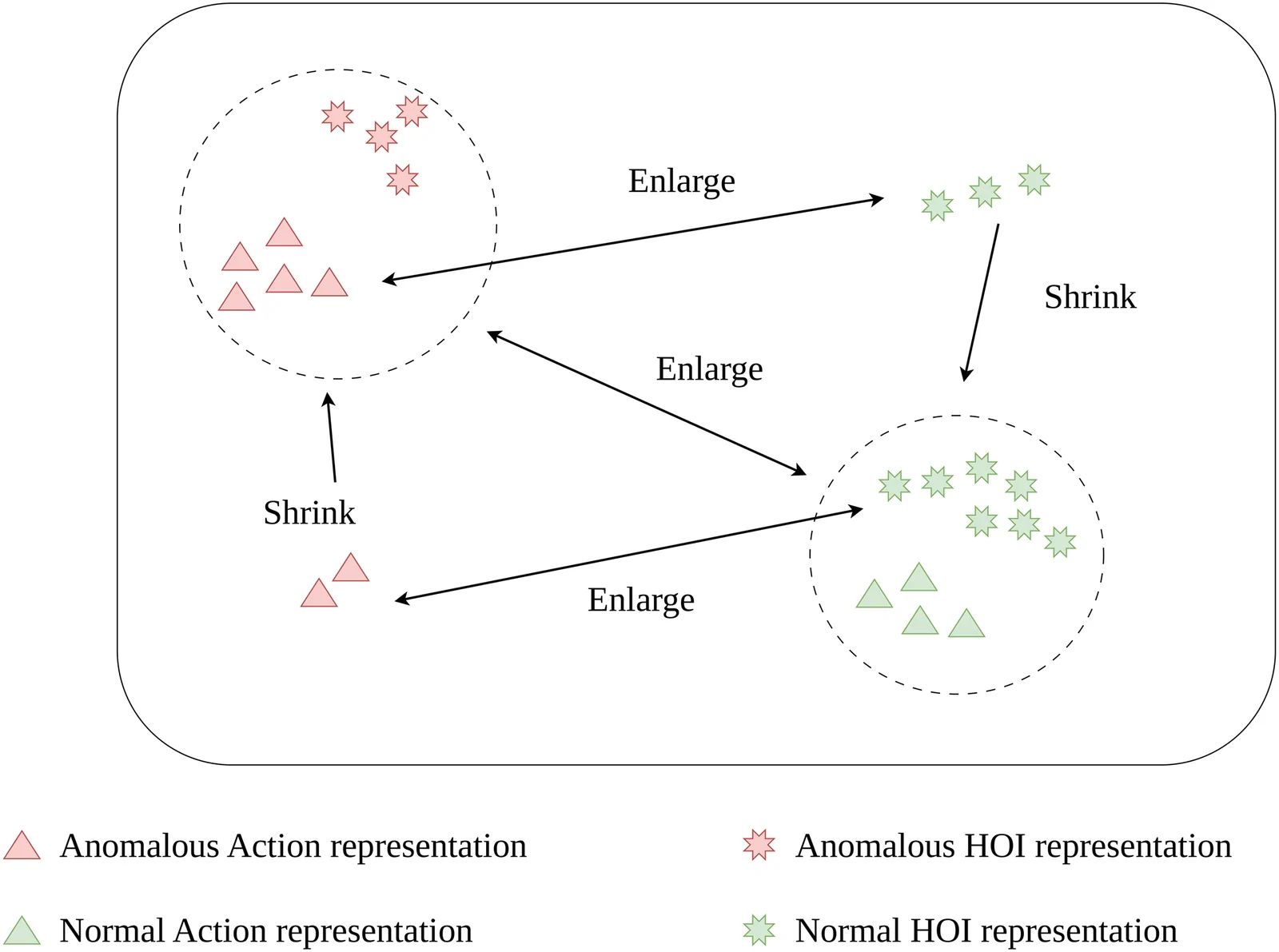
Cross-domain contrastive loss. The purpose of our clip-level cross-domain contrastive (CDC) loss is to learn an adaptive feature magnitude distribution across two domains. It encourages the clustering of intra-class features (i.e., normal or abnormal) across domains while encouraging the separability of inter-class and inter-domain features.

To address this, we introduce a Clip-level Cross-Domain Contrastive (CDC) loss, which is specifically designed to 1) encourage compact intra-class clustering across both domains (action and HOI), and 2) maximize inter-class separation through adaptive distance constraints. Unlike previous works that apply contrastive objectives solely within the action domain [1,23,51], our CDC loss operates across domains and across classes, ensuring that the learned representations are not only robust within each domain but also well-aligned and separable across them, as shown in Fig 4 and Fig 6.

As illustrated in Eq (12), the CDC loss is composed of two components: an intra-class similarity term that clusters features of the same class, and an inter-class contrastive term that enforces a minimum margin between normal and abnormal representations. This formulation allows the model to adaptively balance representation compactness and separability during training, leading to more robust anomaly detection in highly imbalanced and weakly-labeled settings. The ${ℒ}_{cdc}$ is defined as:


$$\begin{array}{c} {ℒ}_{cdc}={ℒ}_{intra}+{ℒ}_{inter}. \end{array}$$ (12)

The intra-class term aggregates pairwise similarities within the same class, computed across both single-domain and cross-domain representations:


$$\begin{array}{c} \begin{array}{cc} {ℒ}_{intra}= & \sum_{i=1}^{\frac{B}{2}}\sum_{j=1}^{\frac{B}{2}}(D(Q_{i}^{n},Q_{j}^{n})+D(X_{i}^{n},H_{j}^{n}))\\ & +\sum_{u=\frac{B}{2}+1}^{B}\sum_{v=\frac{B}{2}+1}^{B}(D(Q_{u}^{a},Q_{v}^{a})+D(X_{u}^{a},H_{v}^{a})), \end{array} \end{array}$$ (13)

where *B* is the batch size, *n* denotes normal video and *a* denotes abnormal video. *Q*, *X*, and *H* represent joint cross-domain, action, and HOI features, respectively.

To enhance the ability of the model to distinguish between normal and abnormal patterns, the inter-class term applies a contrastive margin to penalize between samples from different classes:


$$\begin{array}{c} \begin{array}{cc} {ℒ}_{inter}= & \sum_{i=1}^{\frac{B}{2}}\sum_{u=\frac{B}{2}+1}^{B}(M-D(Q_{i}^{n},Q_{u}^{a}))\\ & +\sum_{i=1}^{\frac{B}{2}}\sum_{u=\frac{B}{2}+1}^{B}(M-D(X_{i}^{n},H_{u}^{a}))\\ & +\sum_{i=1}^{\frac{B}{2}}\sum_{u=\frac{B}{2}+1}^{B}(M-D(H_{i}^{n},X_{u}^{a}))\\ & +\sum_{i=1}^{\frac{B}{2}}\sum_{u=\frac{B}{2}+1}^{B}(M-D(X_{i}^{n},X_{u}^{a}))\\ & +\sum_{i=1}^{\frac{B}{2}}\sum_{u=\frac{B}{2}+1}^{B}(M-D(H_{i}^{n},H_{u}^{a})). \end{array} \end{array}$$ (14)

where D(·,·) denotes the distance function defined in Eq (15), *M* is a margin hyperparameter.

Taking the joint cross-domain features *Q* as an example, we define the pairwise distance between two video clip features:


$$\begin{array}{c} D(Q_{i}^{n},Q_{j}^{n})={\left\|𝐌(Q_{i}^{n})-𝐌(Q_{j}^{n})\right\|}_{2}, \end{array}$$ (15)

where $Q_{i}^{n}=\{q_{i}^{n,t}{\}}_{t=1}^{T}$ and $Q_{j}^{n}=\{q_{j}^{n,t}{\}}_{t=1}^{T}$ denote the clip-level feature sequences of normal videos $V_{i}^{n}$ and $V_{j}^{n}$, with $q_{i}^{n,t},q_{j}^{n,t}\in {\mathbb{R}}^{\frac{D}{4}}$. The feature magnitude vector $𝐌(Q_{i}^{n})$ is defined as:


$$\begin{array}{c} 𝐌(Q_{i}^{n})={\left[\|q_{i}^{n,t}{\|}_{2}\right]}_{t=1}^{T}\in {\mathbb{R}}^{T}, \end{array}$$ (16)

and $𝐌(Q_{j}^{n})$ is defined analogously. The pairwise distances for other feature representations are defined analogously according to Eq (15). This formulation provides a simple yet effective way to measure similarity and separation between clips.

**Clip-level Focal loss** Since the majority of training clips are normal, we leverage clip-level focal loss [34] with generated clip-level abnormal pseudo labels ${\hat{Y}}^{a}$ and clip-level normal labels $Y^{n}$ to mitigate the problem of class imbalance within training clips. The focal loss is an enhanced version of standard binary cross entropy loss, which aims to reduce the relative loss for well-classified frames and focus more on misclassified frames. The focal loss $L_{focal}$ is defined as:


$$\begin{array}{c} {ℒ}_{focal}=\sum_{t=1}^{T}-(\alpha {\hat{y}}_{t}(1-s_{t}{)}^{\gamma }log(s_{t})+(1-\alpha )(1-{\hat{y}}_{t})s_{t}^{\gamma }(log(1-s_{t}))), \end{array}$$ (17)

where α and γ are hyperparameters.

Following previous methods [1,4,6,9], we adopt the sparsity constraint ${ℒ}_{sp}$ and temporal smoothness constraints ${ℒ}_{ts}$ on all predicted snippet scores $f(x^{a})=\{s_{t}^{a}{\}}_{t=1}^{T}$ of the abnormal video clips. ${ℒ}_{sp}$ is defined as:


$$\begin{array}{c} {ℒ}_{sp}=\sum_{t=1}^{T}s_{t}^{a}, \end{array}$$ (18)

and ${ℒ}_{ts}$ is defined as:


$$\begin{array}{c} {ℒ}_{ts}=\sum_{t=1}^{T}(s_{t}^{a}-s_{t+1}^{a}{)}^{2}. \end{array}$$ (19)

The overall loss function is defined as follows:


$$\begin{array}{c} ℒ=\lambda_{1}{ℒ}_{cls}+\lambda_{2}{ℒ}_{cdc}+\lambda_{3}{ℒ}_{focal}+\lambda_{4}{ℒ}_{sp}+\lambda_{5}{ℒ}_{ts}. \end{array}$$ (20)

The weighting coefficients $\lambda_{1-5}$ are empirically determined and remain fixed across all experiments. In practice, we observe that the training process is stable under moderate variations of these weights, as the magnitudes of the different loss terms are comparable during optimization. The overall training procedure of VADHOI is summarized in Algorithm 1.

**Algorithm 1. Training procedure of VADHOI.**

**Input:** Training set $𝒟=\{(F_{action}^{i},F_{hoi}^{i},y_{i},{\hat{Y}}^{i}){\}}_{i=1}^{N}$, batch size *B*, and loss weights $\lambda_{1:5}$

**Output:** Trained model parameters Θ

1: **for** each training iteration **do**

2:  Sample a mini-batch of normal and abnormal videos from 𝒟

3:  Encode the action-domain features: $X\leftarrow E_{action}(F_{action})$

4:  Encode the HOI-domain features: $H\leftarrow E_{hoi}(F_{hoi})$

5:  Fuse the two representations through bi-directional cross-attention:

$Q\leftarrow E_{cross}(X,H)$

6:  Project the joint representation: $Q^{\prime }\leftarrow MLP(Q)$

7:  Model multi-scale temporal dependencies: $Q^{\prime \prime }\leftarrow MTN(Q^{\prime })$

8:  Predict the clip-level anomaly scores: $S=\{s_{t}{\}}_{t=1}^{T}\leftarrow f_{\theta }(Q^{\prime \prime })$

9:  Aggregate the selected clip scores to obtain video-level anomaly scores

10:  Compute ${ℒ}_{cls}$, ${ℒ}_{cdc}$, ${ℒ}_{focal}$, ${ℒ}_{sp}$, and ${ℒ}_{ts}$

11:  Compute the overall loss ℒ according to Eq. (20)

12:  Update Θ by minimizing ℒ

13: **end for**

14: **return** Θ

## Experiments

### Datasets

We demonstrate experimental results on two publicly available benchmarks, i.e., UCF-Crime [6] and ShanghaiTech [17].

UCF-Crime [6] is a large-scale WSVAD benchmark, which covers 13 different types of abnormal events. It contains videos that are recorded in various scenes, such as streets, family rooms, shopping malls, and more. The training set of UCF-Crime contains 1610 videos with coarse video-level labels, and the testing set has 290 videos with precise frame-level labels.

ShanghaiTech [17] is originally proposed for UVAD. The training set contains only normal videos and the abnormal videos only exist in the testing set. It has 437 campus surveillance videos in 13 scenes, 130 of which are abnormal events. Following Zhong et al. [12], the dataset is adapted to the weakly supervised setting by reorganizing the videos such that both the training set and the testing set include abnormal videos, where 238 are used for training and 199 for testing.

### Evaluation metrics

Similar to previous methods [1–11,14] we select the area under the curve (AUC) of the frame-level receiver operating characteristic curve to evaluate the performance of our VADHOI on UCF-Crime and ShanghaiTech. A larger AUC implies a higher distinguishing ability between normal and abnormal events.

### Implementation details

For the MTN, we adopt the same configuration as proposed by Tian et al. [9]. Following previous WSVAD methods [1–4,6,9,10,12,14,15,24,32], both action and HOI features are extracted from every 16 consecutive frames. Since untrimmed videos vary substantially in duration, the resulting feature sequences of each video are averaged to obtain fixed-length representations with *T* clips. The hyperparameters are set as *T* = 32 (number of clips per video) and *D* = 2048 (feature dimension). The number of layers for each encoder component is empirically set to $N_{action}=N_{hoi}=N_{cross}=6$. Following [2], we adopt *k* = 5 as the default setting. In this paper, we use **CLIP-V** to refer to the visual feature embeddings extracted from the visual encoder of CLIP [32]. In contrast, we use the lowercase term *clip* to denote a short temporal segment of a video, typically consisting of a fixed number of consecutive frames (e.g., 16), as commonly adopted in VAD literature.

For the loss functions, we use weights $\lambda_{1}=1$, $\lambda_{2}=0.001$, $\lambda_{3}=0.001$, $\lambda_{4}=0.008$, and $\lambda_{5}=0.0008$. The focal loss parameters are chosen to address the class imbalance during clip-level training: α=0.75 increases the importance of underrepresented abnormal clips, while γ=2 down-weights easy examples to focus learning on harder ones. These values follow the original recommendation in [34] and have been empirically validated to provide stable performance across datasets. For the contrastive loss, we set the margin *M* = 200 following Chen et al. [1].

Optimization is performed using the Adam optimizer [52] with a learning rate of 0.001, batch size of 32, and weight decay of 0.0005. The model is trained for 1500 iterations on a single NVIDIA RTX 4090 GPU.

### Comparison to the state-of-the-art

Table 1 presents the AUC results of our proposed VADHOI framework on the UCF-Crime and ShanghaiTech datasets. It can be observed that VADHOI achieves state-of-the-art performance on ShanghaiTech and highly competitive results on UCF-Crime under the weakly supervised setting. Since our framework is the first to combine human-object interaction (HOI) features with I3D-RGB and **CLIP-V** representations, we report comparisons against prior methods using their officially published results.

**Table 1:** Quantitative comparisons with existing methods on UCF-Crime and ShanghaiTech under different levels of supervision.

Method	Supervised	Features	AUC%	
			UCF-Crime	ShanghaiTech
GODS [53]	one-class	I3D-RGB	70.46	–
Zaheer et al. [22]	one-class	ResNext	74.20	79.62
FRD-UVAD [19]	un	I3D-RGB	82.12	80.14
Liu et al. [5]	full	NLN-RGB	82	–
Sultani et al. [6]	weak	C3D-RGB	75.41	86.30
Sultani et al. [6]	weak	I3D-RGB	77.92	–
Zhang et al. [30]	weak	C3D-RGB	78.66	82.50
MIST [2]	weak	I3D-RGB	82.30	94.83
RTFM [9]	weak	I3D-RGB	84.30	97.21
Wu et al. [11]	weak	I3D-RGB	84.89	97.48
Chang et al. [23]	weak	I3D-RGB	84.62	92.25
MSL [3]	weak	I3D-RGB	85.30	96.08
MSL [3]	weak	VideoSwin-RGB	85.62	97.32
S3R [10]	weak	I3D-RGB	85.99	97.48
SSRL [4]	weak	I3D-RGB	87.43	97.98
MGFN [1]	weak	I3D-RGB	86.98	–
MGFN [1]	weak	VideoSwin-RGB	86.67	–
ARMS [7]	weak	I3D-RGB	85.79	97.48
UR-DMU [24]	weak	I3D-RGB	86.97	–
Clip-tsa [32]	weak	**CLIP-V**	87.58	98.32
Tan et al. [8]	weak	I3D-RGB+Flow	86.71	97.54
BN-WVAD [13]	weak	I3D-RGB	87.24	97.61
PEL4VAD [14]	weak	I3D-RGB	86.76	98.14
VadCLIP [31]	weak	**CLIP-VL**	**88.02**	–
VADHOI(ours)	weak	I3D-RGB + HOI	87.81	97.66
VADHOI(ours)	weak	**CLIP-V** + HOI	87.96	**98.33**

Bold represents the best results. - means there is no official result on the ShanghaiTech dataset. **CLIP-V** denotes visual-only features extracted from the CLIP visual encoder, whereas **CLIP-VL** represents joint vision-language features derived from both the visual and text encoders of CLIP.

Specifically, VADHOI achieves an AUC of **87.96%** on UCF-Crime, outperforming previous one-class UVAD methods such as GODS [53], Zaheer et al. [22], and the unsupervised FRD-UVAD [19]. It also exceeds the fully supervised approach of Liu et al. [5]. Compared with existing weakly-supervised methods, VADHOI shows clear performance advantages. It surpasses RTFM [9] by 3.66%, MSL [3] using I3D-RGB by 2.66%, MSL with VideoSwin-RGB by 2.34%, and S3R [10] by 1.97%. It further improves upon SSRL [4] (87.43%), MGFN [1] (86.98%), ARMS [7] (85.79%), UR-DMU [24] (86.97%), Clip-tsa [32] (87.58%), Tan et al. [8] (86.71%), BN-WVAD [13] (87.24%), and PEL4VAD [14] (86.76%). Although VADCLIP [31] achieves a slightly higher AUC of 88.02%, it relies on both the image and text branches of CLIP for cross-modal alignment. In contrast, VADHOI achieves comparable performance using only purely visual features that do not rely on any textual or prompt-based guidance.

On the ShanghaiTech dataset, VADHOI achieves an AUC of **98.33%**, outperforming all existing methods. It exceeds MIST [2] by 3.50%, RTFM [9] by 1.12%, S3R [10] and ARMS [7] by 0.85%, SSRL [4] by 0.35%, Clip-tsa [32] by 0.01%, Tan et al. [8] by 0.79%, BN-WVAD [13] by 0.72%, and PEL4VAD [14] by 0.19%. The relatively smaller improvement margin on ShanghaiTech is expected due to its simpler anomaly types and limited interactive complexity. Nevertheless, these results confirm the effectiveness of our cross-domain framework, which integrates both motion and interaction cues to enhance anomaly discrimination, particularly in complex real-world surveillance scenarios.

### Ablation studies

Ablation studies were conducted to analyze the effectiveness of each key component of our proposed framework: cross-domain learning module, cross-domain contrastive loss, and clip-level focal loss.

**Ablation Studies of HOIAL.** To evaluate the individual contributions of each component within the proposed HOIAL module, we conduct ablation studies on the UCF-Crime and ShanghaiTech datasets using CLIP-V as the action feature extractor. The results are presented in Table 2. We construct three baselines by progressively enabling different combinations of the encoders in HOIAL: (1) using only the action encoder $E_{action}$, (2) using only the HOI encoder $E_{hoi}$, and (3) using both $E_{action}$ and $E_{hoi}$ without the cross-domain encoder $E_{cross}$. The final row shows the full VADHOI model with all three components enabled. From the results, we observe that using either single-domain encoder independently provides reasonable performance (86.83% and 85.99% on UCF-Crime), while their combination without cross-domain fusion actually leads to a slight degradation (83.58%), likely due to feature misalignment. However, when the cross-domain encoder is included, our model achieves the best results: 87.96% on UCF-Crime and 98.33% on ShanghaiTech. Notably, the performance gain on UCF-Crime is more pronounced than on ShanghaiTech. This aligns with the greater semantic complexity and diversity of real-world anomalies in UCF-Crime, indicating that the proposed cross-domain learning is especially beneficial for challenging surveillance scenarios.

**Table 2:** Ablation studies of our HOIAL module with CLIP-V on UCF-Crime and ShanghaiTech.

$E_{action}$	$E_{hoi}$	$E_{cross}$	UCF-Crime (AUC%)	ShanghaiTech (AUC%)
✓			86.83	97.92
	✓		85.99	97.76
✓	✓		83.58	97.41
✓	✓	✓	**87.96**	**98.33**

**Ablation Studies of HOIAL Module on different visual backbones.** To verify the effectiveness and generality of the proposed HOIAL module across different visual encoders, we conduct ablation studies using two types of action features: I3D and **CLIP-V** [32]. As shown in Table 3, we compare the performance of our method with and without HOIAL on the UCF-Crime and ShanghaiTech datasets. When using I3D-RGB features, incorporating HOIAL leads to a substantial performance gain. Specifically, the AUC improves from 84.03% to 87.81% on UCF-Crime and from 96.13% to 97.66% on ShanghaiTech. This demonstrates that the integration of HOI semantics with action features enhances anomaly discrimination, especially in the more complex and diverse video scenarios of UCF-Crime. Even with **CLIP-V** features, which already encode rich visual semantics due to vision-language pretraining, HOIAL consistently improves performance. The AUC increases from 85.85% to 87.96% on UCF-Crime and from 97.93% to 98.33% on ShanghaiTech. Although the improvement margins are smaller compared to I3D, these results confirm that HOIAL can effectively complement both traditional and pre-trained feature extractors. Overall, the consistent improvements across different visual backbones validate the robustness and adaptability of the HOIAL module for cross-domain anomaly detection tasks.

**Table 3:** Ablation studies of HOIAL module on different visual backbones.

Baseline	Visual backbone	HOIAL	UCF-Crime (AUC%)	ShanghaiTech (AUC%)
✓	I3D-RGB		84.03	96.13
✓	I3D-RGB	✓	87.81 (↑3.78)	97.66 (↑1.53)
✓	**CLIP-V**		85.85	97.93
✓	**CLIP-V**	✓	**87.96** (↑2.11)	**98.33** (↑0.40)

“Baseline” means VADHOI without HOIAL module.

**Ablation studies of Cross-Domain Contrastive Loss and Clip-Level Focal Loss.** We conduct ablation studies to evaluate the contribution of the cross-domain contrastive loss ${ℒ}_{cdc}$ and the clip-level focal loss ${ℒ}_{focal}$, as summarized in Table 4. The baseline model in this setting refers to VADHOI with the HOIAL module but without the two loss components. Introducing only ${ℒ}_{cdc}$ leads to a noticeable improvement in AUC, from 86.68% to 87.28% on UCF-Crime and from 97.34% to 98.12% on ShanghaiTech. This confirms the effectiveness of aligning and separating cross-domain features to enhance anomaly discrimination. Similarly, adding only ${ℒ}_{focal}$ increases the AUC to 87.06% on UCF-Crime and 97.97% on ShanghaiTech, indicating that emphasizing hard and minority abnormal samples at the clip level benefits training stability and detection sensitivity. When both losses are applied together, our framework achieves the best results: 87.96% on UCF-Crime and 98.33% on ShanghaiTech, corresponding to absolute improvements of 1.28% and 0.99% over the baseline. These findings validate the complementary nature of ${ℒ}_{cdc}$ and ${ℒ}_{focal}$ in enhancing the representational power of our model.

**Table 4:** Ablation studies of proposed losses on UCF-Crime and ShanghaiTech.

Baseline	${ℒ}_{cdc}$	${ℒ}_{focal}$	UCF-Crime (AUC%)	ShanghaiTech (AUC%)
✓			86.68	97.34
✓	✓		87.28	98.12
✓		✓	87.06	97.97
✓	✓	✓	**87.96** (↑1.28)	**98.33** (↑0.99)

“Baseline” means VADHOI with HOIAL, without ${ℒ}_{cdc}$ and ${ℒ}_{focal}$.

**Ablation Studies of MTN.** We further analyze the impact of MTN. Specifically, we replace MTN with a simple fully connected (FC) temporal layer while keeping all other components unchanged. As shown in Table 5, MTN achieves higher AUC scores than the FC temporal layer on both datasets, demonstrating the advantage of capturing both long-range and short-range temporal dependencies at multiple scales.

**Table 5:** Ablation studies of MTN on UCF-Crime and ShanghaiTech.

Temporal Modeling	UCF-Crime (AUC%)	ShanghaiTech (AUC%)
FC layer (w/o MTN)	86.54	97.18
MTN (ours)	**87.96**	**98.33**

**Sensitivity to Temporal Smoothing Kernel Size in PLG.** We analyze the sensitivity of the temporal smoothing kernel size *k*. As shown in Table 6, the performance is stable across different values of *k* from 3 to 9, with AUC variations within 0.2% on both datasets. Although *k* = 5 achieves slightly higher performance, neighboring settings yield comparable results, indicating that the proposed method is robust to the choice of kernel size.

**Table 6:** Ablation studies of the temporal smoothing kernel size *k* on UCF-Crime and ShanghaiTech.

*k*	UCF-Crime (AUC%)	ShanghaiTech (AUC%)
3	87.83	98.14
5	**87.96**	**98.33**
7	87.91	98.23
9	87.78	98.19

**Ablation studies of thresholding strategies in PLG.** We also investigate the influence of different thresholding strategies in PLG. Specifically, we compare mean-based and median-based thresholding applied independently within each abnormal video. As shown in Table 7, the performance difference between the two strategies is marginal on both datasets. Although mean-based thresholding achieves comparable AUC scores, it results in a substantially higher proportion of clips being labeled as abnormal (approximately 84.6% within abnormal videos). In contrast, median-based thresholding yields a more moderate distribution of pseudo labels, avoiding an overwhelming majority of positive assignments. Since the anomaly scores predicted by PLG may vary in scale across different videos, the median serves as a video-adaptive decision boundary that preserves the relative ranking of clips within each video. This prevents excessive dominance of positive pseudo labels and leads to more stable optimization of the focal loss.

**Table 7:** Ablation studies of the thresholding strategies on UCF-Crime and ShanghaiTech.

Threshold strategy	UCF-Crime (AUC%)	ShanghaiTech (AUC%)
mean-based	87.67	98.09
median-based	**87.96**	**98.33**

**Model Size, Speed, and Computational Complexity.** As shown in Table 8, VADHOI achieves a favorable balance between model size and computational complexity when compared to other state-of-the-art methods. With a parameter count of 53.84M, our model remains moderately sized—significantly smaller than SSRL [4] (191M) and Zhong et al. [12] (78M). More importantly, VADHOI exhibits notably low computational overhead, with only 2.48 GFLOPs, which is substantially lower than high-complexity baselines such as MIST [2] (45.68G), SSRL [4] (214.6G), and Zhong et al. [12] (386.2G). Compared to lightweight methods like RTFM [9] (0.79G) and ARMS [7] (0.34G), VADHOI offers significantly stronger detection performance while maintaining acceptable computational cost. Moreover, our framework requires less than 1 ms to compute frame-level anomaly scores on a single RTX 4090.

**Table 8:** Comparison of model size and computational complexity.

Method	#Params	#FLOPs
Zhong et al. [12]	78M	386.2G
MIST [2]	31M	45.68G
RTFM [9]	24.72M	0.79G
SSRL [4]	191M	214.6G
ARMS [7]	10.53M	0.34G
PEL4VAD [14]	1.21M	241M
VADHOI(ours)	53.84M	2.48G

Although the proposed VADHOI framework introduces additional components for HOI feature integration and cross-domain attention, the resulting computational overhead remains limited. Specifically, the HOI representations are obtained using an off-the-shelf detector and incorporated as high-level semantic features, while the cross-domain attention module mainly consists of lightweight attention operations applied to pre-extracted visual features. Therefore, these components do not require expensive end-to-end detection during training or inference. In practice, the overall runtime is still dominated by the backbone visual feature extraction stage, and the proposed modules introduce only a modest increase in memory usage and computation. This design enables the framework to enhance semantic modeling while maintaining practical computational efficiency. These results demonstrate that VADHOI strikes an effective trade-off between accuracy, model compactness, and inference efficiency.

### Qualitative results

**Fig 5:**
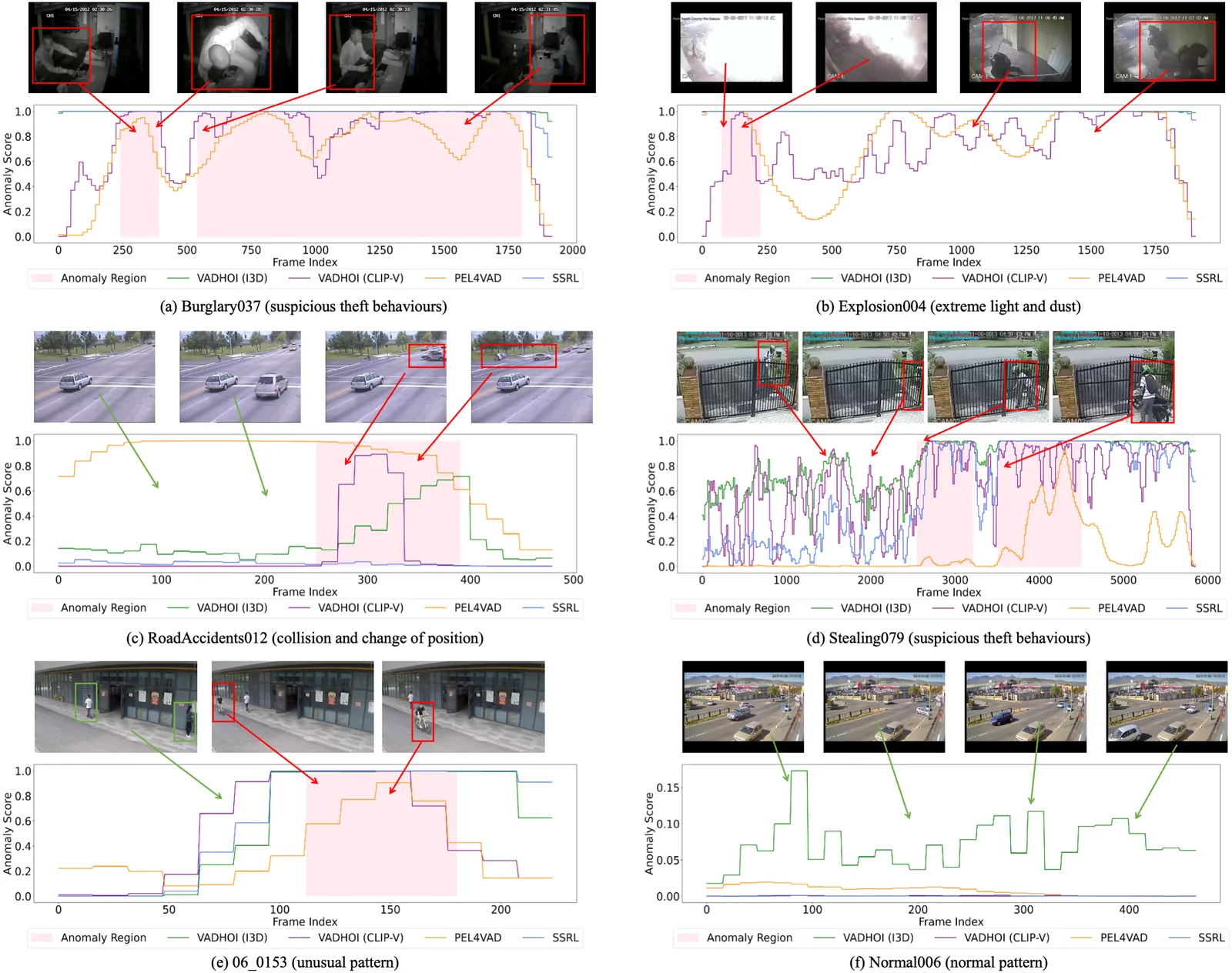
Qualitative visualization of the predicted anomaly scores. The scores are produced by the proposed VADHOI framework, SSRL [4], and PEL4VAD [14] on the UCF-Crime dataset (Burglary037, Explosion004, RoadAccidents012, Stealing079, Normal006) and the ShanghaiTech dataset (06_0153). The pink regions indicate the ground-truth temporal locations of abnormal events.

**Qualitative visualization of predicted anomaly scores.** To comprehensively evaluate the effectiveness and generalizability of the proposed VADHOI framework, we visualize the predicted frame-level anomaly scores on a set of videos from two benchmark datasets: five videos from UCF-Crime (including four abnormal and one normal video) and one abnormal video from ShanghaiTech, as illustrated in Fig 5. Although VADHOI is primarily designed to incorporate human-centric interaction features, it remains highly competitive in detecting both human-involved and non-human-centric anomalies (e.g., Explosion, RoadAccidents). For instance, in video Burglary037, VADHOI produces a sharp and temporally precise anomaly score that aligns closely with the suspicious interactions, outperforming baselines such as SSRL [4] and PEL4VAD [14], which exhibit delayed or smoothed responses. Notably, in video Stealing079, VADHOI anticipates the anomalous event by assigning elevated scores to suspicious pre-event behaviors, such as repeated approaches to a gate and close-proximity human-object interactions, highlighting the capability of HOI modeling to capture precursor cues effectively.

More importantly, VADHOI also performs well in non-human-centric scenarios where HOI signals are weak or even absent. In videos such as Explosion004 and RoadAccidents012, the anomalies are mainly driven by global scene dynamics rather than explicit human–object interactions, yet the model accurately localizes the anomalous segments with minimal false positives. In some cases (e.g., Explosion004, RoadAccidents012, and Stealing079), the detected HOI cues may be weak or partially irrelevant due to the nature of the events or subtle interactions, which may introduce noise into the anomaly scores. Nevertheless, the proposed framework still maintains reliable anomaly localization by integrating HOI representations with complementary action features and temporal modeling. These observations suggest that incorporating HOI features does not limit the applicability of the framework. While HOI enriches semantic reasoning in complex, human-involved scenes, the overall design generalizes effectively to a wide range of anomaly types, including vehicle accidents, environmental hazards, and other scene-level anomalies. Moreover, for the normal video Normal006, VADHOI maintains consistently low anomaly scores, demonstrating its robustness in suppressing false positives under normal conditions.

**Fig 6:**
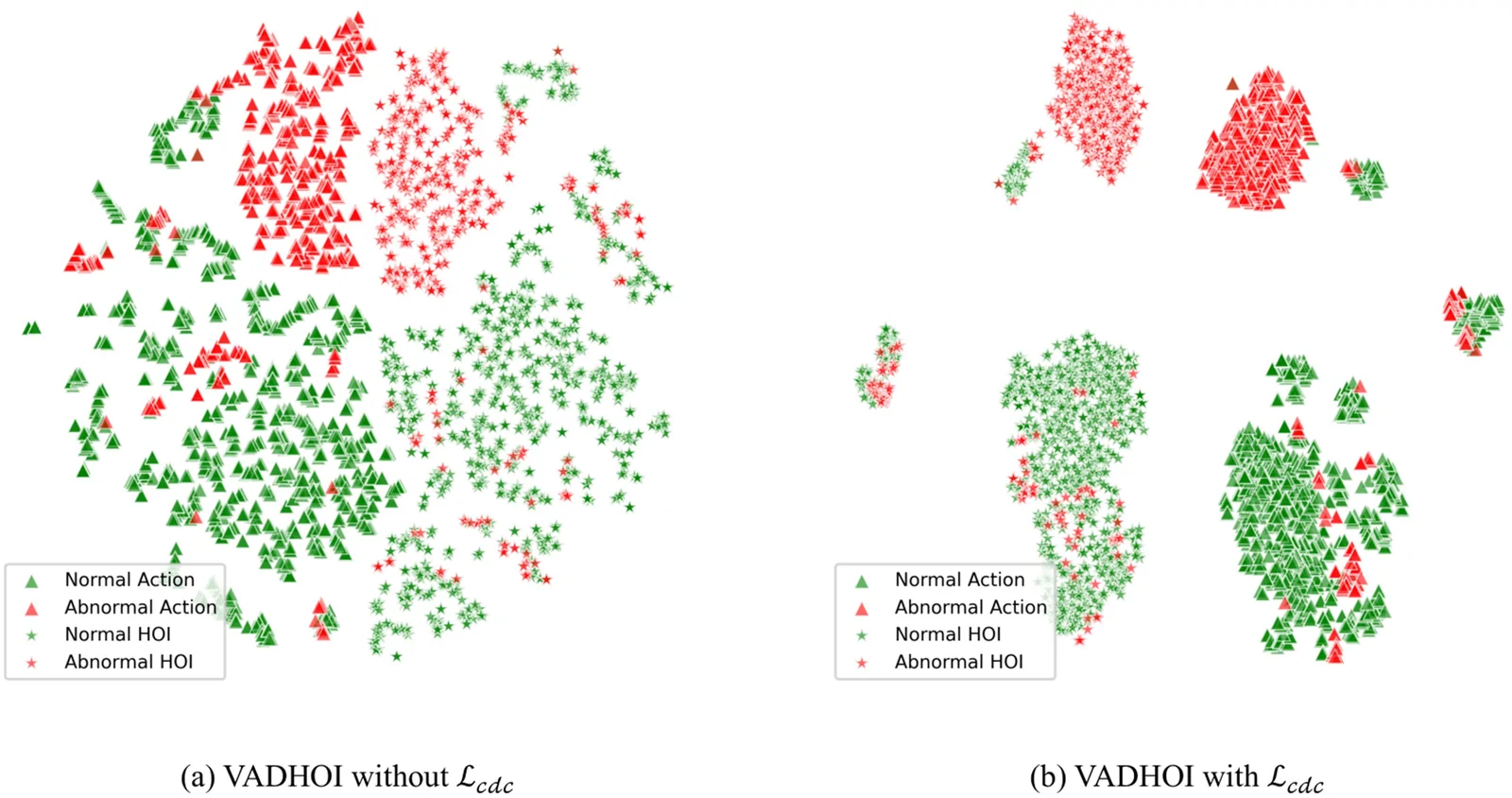
Feature distribution of the cross-domain features without and with ${ℒ}_{cdc}$ using t-SNE on UCF-Crime.

**Feature distribution.** To further demonstrate the effectiveness of the proposed cross-domain contrastive loss ${ℒ}_{cdc}$, we visualize the distribution of cross-domain features using t-SNE on the UCF-Crime dataset, as illustrated in Fig 6. In Fig 6(a), where ℒcdc is not applied, features from different classes (normal vs. abnormal) and domains (action vs. HOI) exhibit relatively poor discriminability. In contrast, Fig 6(b) shows that incorporating ℒcdc results in clearly separated clusters, with improved intra-class compactness and inter-class separability across both action and HOI features. These results confirm that ${ℒ}_{cdc}$ effectively enhances the discriminative capacity of cross-domain representations, particularly under severe class imbalance.

## Discussion

While the proposed VADHOI framework primarily focuses on HOI, the cross-domain design that integrates HOI representations with action semantics allows the model to leverage complementary visual cues and remain effective across diverse anomaly scenarios. Nevertheless, several limitations remain. Since the framework leverages pre-trained HOI detectors to extract interaction features, the quality of the HOI representations may be affected when interaction predictions are noisy or incomplete. This issue may become more pronounced in complex scenes, such as highly crowded environments with occlusions and overlapping activities, where reliable interaction detection is inherently difficult. In addition, anomalies primarily driven by global contextual changes rather than localized interactions may also provide weaker HOI cues, potentially introducing uncertainty into the interaction representations. Although the proposed cross-domain fusion and multi-scale temporal modeling mechanisms help mitigate these issues by incorporating complementary visual features, further improving the robustness of interaction modeling and incorporating richer scene-level context remain promising directions for future work.

## Conclusion

Although many existing anomaly detection datasets are biased toward crime-related or human-centric events, our results demonstrate that VADHOI is not inherently constrained by this bias. The framework exhibits strong performance across both interaction-dense and interaction-sparse scenarios, highlighting its practicality for real-world deployments involving a wide variety of anomalous behaviors. While our framework primarily focuses on human-object interactions, the cross-domain design of combining HOI and action semantics already enables generalization to anomalies without explicit human involvement, such as explosions or road accidents. In future work, we aim to extend the framework to incorporate more general object-object and scene-level interactions, thereby enhancing its robustness in detecting complex, non-human-centric anomalies. We hope this work will inspire further research into semantically enriched, interpretable, and ethically responsible VAD systems.
